# Complications and survival rates of subtrochanteric fractures are similar between short and long intramedullary nail fixation and independent of weight-bearing

**DOI:** 10.1007/s00590-024-03992-w

**Published:** 2024-05-21

**Authors:** Daniel Cohen, Yaakov Tolwin, Michael Toybenshlak, Gershon Zinger, Amos Peyser, Yadin Levy

**Affiliations:** Department of Orthopedic Surgery, Shaare Zedek Medical Center, 12 Shmuel Bait St, PO Box 3235, 9103102 Jerusalem, Israel

**Keywords:** Subtrochanteric, Weight-bearing, Nail length

## Abstract

**Purpose:**

Intertrochanteric fractures are treated surgically, allowing rapid weight-bearing to improve ambulation and lower complications and mortality. Subtrochanteric fractures are mechanically less stable and are traditionally treated with a non-weight approach and longer intramedullary nails. This study compared immediate weight-bearing versus limited weight-bearing and different intramedullary nail lengths regarding patient outcomes.

**Methods:**

We analyzed all consecutive cases of low-energy subtrochanteric fractures treated surgically at our institution between January 2016 and November 2020. One hundred and nine patients were found. We compared nail length and immediate versus delayed weight-bearing concerning the length of stay, time to painless ambulation, time to radiographic fracture union, and revision rates. Fracture severity was also examined using the Seinsheimer classification.

**Results:**

Length of stay and time to painless ambulation were shorter in the immediate weight-bearing group. Time to radiographic union and rate of complications were lower; however, they were not statistically significant. Conversely, no significant difference in revision rates was found. Regarding nail length, the length of stay was shorter, and the time to painless ambulation was faster in the short-length group. The rate of complications and time to union were similar. No difference in revision rate was found. Seinsheimer classification of the fracture did not influence the decision to allow weight-bearing or nail selection (*p* = 0.65).

**Conclusions:**

This study demonstrates that immediate weight-bearing as tolerated and short intramedullary nails allow a quicker time for painless ambulation and hospitalization, with possibly fewer perioperative complications and faster radiographic union, without increasing complications.

## Introduction

Subtrochanteric femur fractures in the elderly are primarily osteoporotic fractures that result from low-energy falls [1]. These fractures are traditionally treated with a long cephalon-medullary nail [2], with restricted weight-bearing for approximately six weeks before progressing to full weight-bearing [3]. Several recent studies have suggested that patients with these fractures may be allowed immediate weight-bearing, like intertrochanteric fractures [4]. Immediate weight-bearing after fractures, in general, has been shown to reduce the risk of mortality and perioperative complications while allowing faster rehabilitation [5], including a recent study regarding subtrochanteric fractures specifically [6]. However, subtrochanteric fractures are considered relatively unstable [7] and, therefore, not commonly allowed to bear weight.

Simultaneously, these fractures are commonly treated with a long (beyond isthmus) intramedullary or cephalo-medullary nail due to the inherent instability [3]. In general, short and medium intramedullary nails are preferable due to the shorter surgical time and decreased surgical blood loss, utilizing a dedicated guide for positioning the distal locking screw, which is unavailable with long nails [8, 9]. Additionally, due to the bowing of the femur, long nails usually require extended reaming and could have more complications during insertion [10]. Nonetheless, long nails are traditionally considered safer as they protect the entire length of the femur [11]. Perhaps due to the additional complications, some studies have shown extended hospital stay (LOS) with long nails [12].

Focusing on subtrochanteric fractures in elderly patients, study endpoints included the length of hospital stay, time to painless ambulation, time to radiographic fracture union, revision rates, and perioperative medical complications. This study aims to show that early weight-bearing and short-nail results are not worse than those achieved with delayed weight-bearing and long nails and could even be better. Hence, the current treatment protocols might be revised, enabling patients to undergo a shorter surgery, with no reaming sequela, and to be able to bear weight earlier.

## Materials and methods

A database file was created by the hospital records department of all patients presenting to our level 1 trauma center with subtrochanteric fractures of the femur, based on the ICD9 code, between January 2016 and November 2020. We excluded cases under age 60, periprosthetic fractures, patients treated conservatively, and patients transferred to other hospitals before surgery. The study included 109 patients. Patient characteristics are listed in Table 1.

**Table 1:** Demographic data of the cohorts

	WBAT	DB	*P* value	Standard (< 235 mm)	Long (> 235 mm)	*P* value
Number of Pts	75	34	NA	40	69	NA
Age (avg.)	82.6	81.5	0.56	84.2	80.9	0.06
Fracture parts (avg.)	2.85	2.51	** < 0.05**	2.85	2.72	0.32
ASA score (avg.)	2.5	2.65	0.22	2.61	2.52	0.45

Bold value is statistically significant (*p* < 0.05)

**Fig. 1:**
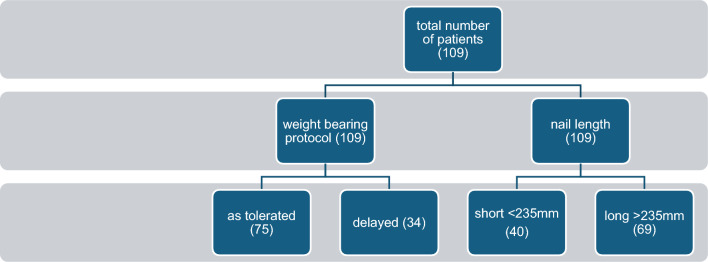
Flowchart of cohort division

Regarding the weight-bearing protocol, patients were divided into two groups based on post-op mobility recommendations (Fig. 1). The weight-bearing as tolerated (WBAT) cohort included all patients who were instructed to fully bear weight as endured, using any mobility assistance devices they or the physical therapy team felt necessary. This protocol was instated immediately post-surgery. The delayed weight-bearing (DB) group included patients instructed not to fully bear weight immediately after surgery but to limit weight-bearing to transitions only or to practice minimal protected weight-bearing, assisted by a walker. In cases where the recommendations were not explicit, the physical therapy notes, postoperative medical follow-up, and surgical notes were used to determine the weight-bearing protocol recommended to the patient. The rehabilitation was initiated while hospitalized and continued on at the rehabilitation facilities.

Regarding nail length—patients were divided into two groups based on the length of the nail used (Fig. 1): The short-length group includes all fractures fixed with a nail not passing the femoral isthmus. The long-nail group had all fractures fixed with isthmus-spanning nails, reaching at least the metaphyseal region of the distal femur.

The cohorts were compared regarding the length of hospital stay, time to painless ambulation, time to radiographic fracture union, revision rates, and perioperative medical complications. These complications include respiratory difficulty, urinary complaints, and coagulopathy, among others. When calculating revision rates, we excluded revision surgery due to additional trauma and immediate revision due to iatrogenic causes (implant malpositioning, limb malrotation). The post-surgical protocol included a medical examination with pelvic/femur radiographs—1.5/3/6/12/24/36 months post-op. Radiographic union, which demonstrated cortical continuity, was noted based on patient X-rays in the clinic or clinic notes when patient X-rays shown in the clinic were unavailable. The time to radiographic union is the first X-ray showing fracture union. Time to painless ambulation was calculated based on clinical notes. Medical complications were defined as non-orthopedic complications within 30 days of surgery, resulting in the need for patient transfer from the orthopedic ward during the hospitalization or a need for emergency ward evaluation.

**Fig. 2:**
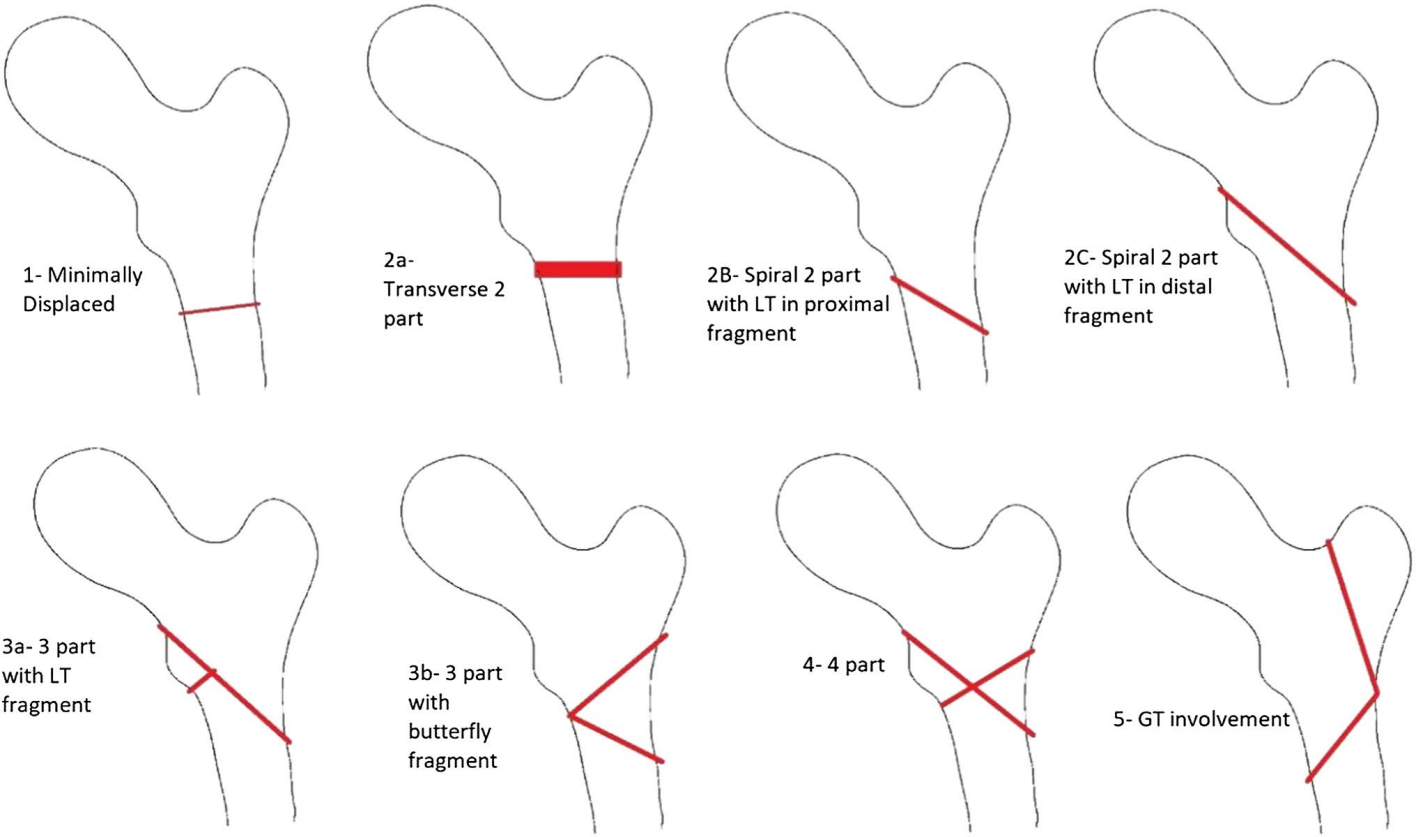
Seinsheimer classification

Seinsheimer classification was used to assess fracture severity [13] (Fig. 2). While no classifications for subtrochanteric fractures have been shown to impact treatment [14], and the Seinsheimer classification was shown to have poor interobserver reproducibility [12], it is seen as the classification best describing subtrochanteric fractures [4]. The classification was used to appreciate that more severely comminuted fractures would be less stable after fixation and more likely to fail with immediate weight-bearing.

The Institutional Research Ethics Committee has approved this observational study, which did not require specific patient consent.

## Statistical methods

Statistical calculations were performed using the two-sample T-test for independent means and continuous variables. The chi-square test for goodness of fit was used to compare categorical datasets. The correlation between Seinsheimer classification and increased likelihood of limited weight-bearing was examined with R^2^.

Statistical significance was defined as a *P* value < 0.05.

## Results

Patient characteristics did not significantly differ between both groups Table 1.

### Weight-bearing results

The average length of hospital stay was 9.9 days WBAT versus 11.6 days DB (P-value 0.054). The average time to painless ambulation was 4.7 months WBAT versus 10.4 DB (P-value < 0.01). The average time to radiographic union was 7.5 months WBAT versus 8.2 DB (P-value 0.65). The revision rate, due to hardware/fixation failure, was 9% WBAT versus 14% DB (*P*-value = 0.4). The rate of medical complications was 14% WBAT versus 26% DB (P-value = 0.14). Seinsheimer classification did not significantly correlate with limited weight-bearing, with an *R*^2^ value of 0.0002. The average combined follow-up time was 7.93 months—6.9 months for the WBAT group and 11.7 months for the DB group. Results are summarized in Table 2.

**Table 2:** Comparison of functional results between both study cohorts

*P*	WBAT	DB	*P* value	Short-nail group	Long-nail group	*P* value
Average length of hospital stay	9.9	11.6	**0.054**	9.4	10.8	**0.05**
Average time to painless ambulation	4.7	10.4	** < 0.01**	4.3	6.7	**0.05**
Average time to radiographic union	7.5	8.2	0.65	6.8	7.3	0.67
Revision rate	9/75 (12%)	14/34 (41.2%)	0.2	5/40 (12.5%)	8/69 (11.5%)	0.45
Follow-up (months on avg.)	6.4	7.9	0.08	5.26	7.96	***p* < 0.05**

Bold values are statistically significant (*p* < 0.05)

### Nail length

The average length of hospital stay was 9.4 (short) versus 10.8 days (long) (*P* < 0.05). The average time to painless ambulation was 4.3 months (short) versus 6.7 (long) (*P* < 0.05). The average time to radiographic union was 6.8 months (short) versus 7.3 (long) (*P*= 0.67). The revision rate was 12.5% (short) versus the 11.5% (long) group (*P* = 0.88). The rate of medical complications was 20% (short) versus 17.4% (long) (*P* = 0.73). Long nails did not significantly correlate with the Seinsheimer classification, with an *R*^2^ value of 0.179. Results are summarized in Table 2.

## Discussion

While immediate full weight-bearing is common practice for intertrochanteric fractures after nail fixation and has been shown to offer many benefits to patients, subtrochanteric fractures are typically not allowed full weight-bearing due to the dissimilar biomechanics of the fracture and risk for fixation failure. Recent studies have shown that full weight-bearing after fixation of subtrochanteric fractures allows for shorter hospitalization times and low rates of implant failure in certain groups of patients [4, 6]. However, minimal data regards the longer-term effect of immediate weight-bearing, particularly in the elderly, including the risk of developing perioperative medical complications. This study was designed to examine the impact of immediate weight-bearing and short-nail practice on subtrochanteric fractures in the geriatric population in terms of length of stay and the length of time to fracture union, painless ambulation, rate of non-orthopedic complications, and revision surgery.

Immediate weight-bearing as tolerated after geriatric subtrochanteric fracture fixation allowed a shorter time to painless ambulation, which is most likely a result of preserving muscle strength, otherwise impaired due to extended bed rest. The study also showed that immediate weight-bearing resulted in fewer perioperative complications and shorter hospital stays while not increasing the need for revision surgeries. Earlier weight-bearing hastened the union rate due to the increased forces on the fracture. Nonetheless, age, ASA score, and the Seinsheimer classification did not significantly differ between the cohorts, meaning that the complexity of the fracture nor the patients’ medical background were meaningful and, therefore, should not be relevant for the weight-bearing decision.

Cephalo-medullary nails are commonly used for the fixation of femoral subtrochanteric fractures. Short nails are common for intertrochanteric fractures, offering many benefits to patients, such as shorter operative time and less perioperative bleeding. Subtrochanteric fractures are commonly fixed with long nails due to the different biomechanics of the fracture, possibly compromising fixation stability [15]. The distal screw targeting device is not accurate enough in the longer nails, necessitating a “free hand” method. Besides additional surgical time, the nail might be missed by the screw, causing further cortical punctures or even an unlocked device. The natural bowing of the femur causes a mismatch with the longer nails. Hence, for insertion, medullary reaming is often needed. Besides increasing surgical time, reaming might cause systemic complications such as fat emboli and additional bleeding.

Looking at similar cohorts, short cephalo-medullary nails for geriatric subtrochanteric fracture fixation allowed a shorter time for painless ambulation. They possibly allowed shorter hospital stays and time to union, likely due to the shorter surgical time and decreased need for reaming with short nails. While the study also showed that short nails possibly resulted in more perioperative complications and increased the demand for revision surgeries, the differences were minor and not statistically significant.

As Table 3 demonstrates, surgeons in our facility did not allow weight-bearing in most cases when using long nails. They, too, were under the popular concept that using a long nail requires the absence of weight-bearing to reduce the likelihood of fixation failure. Orthopedic hardware information regarding the nails is mentioned as well.

**Table 3:** Fracture fixation data

*P*	WBAT	DB	*P* value
Long nail (> 235 mm)	41 (55%)	27 (80%)	** < 0.05**
Short nail (<235 mm)	34 (45%)	7 (20%)	
Gamma3 (Stryker)	60 (80%)	26 (76%)	0.67
TFNA (Synthes)	6 (8%)	2 (6%)	
Other	9 (12%)	6 (18%)	

Bold value is statistically significant (*p* < 0.05)

One concern for this study is the cohort size, which needed to be more adequately powered to show the significance of some of the results. Further research with larger patient cohorts would offer added benefits, allowing for further stratification to determine which patients would most likely benefit from early mobilization. Also, some results are based on clinical follow-up visits, and the time between visits impacts these results. An additional weakness is the high number of patients lost to follow-up, which, unfortunately, is common among elderly patients. Moreover, studies have shown that elderly patients do not always follow weight-bearing protocols, particularly concerning assisted or protected weight-bearing instructions. Despite these weaknesses, this study indicates the non-inferiority of immediate weight-bearing and short-nail usage in these clinical settings.

## Conclusion

Immediate weight-bearing as tolerated, together with short intramedullary nails, allows a shorter time to painless ambulation, possibly fewer perioperative complications, and shorter hospital stays without an increased implant failure rate. Given the well-established benefits in the geriatric population, we recommend early weight-bearing and short intramedullary nails when possible. However, when making this decision, surgeons must consider the specific fracture characteristics and the attained stability of the fixation.

## Data Availability

(Data transparency)—Not applicable.

## Code availability

(Software application or custom code)—Not applicable.
